# Three-Dimensional Quantitative Structure-Activity Relationships (3D-QSAR) on a Series of Piperazine-Carboxamides Fatty Acid Amide Hydrolase (FAAH) Inhibitors as a Useful Tool for the Design of New Cannabinoid Ligands

**DOI:** 10.3390/ijms20102510

**Published:** 2019-05-21

**Authors:** Marcos Lorca, Yudisladys Valdes, Hery Chung, Javier Romero-Parra, C. David Pessoa-Mahana, Jaime Mella

**Affiliations:** 1 Escuela de Quimica y Farmacia, Facultad de Medicina, Universidad Andres Bello, Quillota 980, Viña del Mar 2531015, Chile; m.lorcacarvajal@uandresbello.edu; 2 Pharmacy Department, Faculty of Chemistry, Pontificia Universidad Católica de Chile, Vicuña Mackenna 4860, Santiago 7820436, Chile; ylvaldes@uc.cl (Y.V.); chung.hery@gmail.com (H.C.); 3 Departamento de Ciencias Farmacéuticas, Facultad de Ciencias, Universidad Católica del Norte, Avenida Angamos 0610, Antofagasta 1270709, Chile; javier.romero@ucn.cl; 4 Instituto de Química y Bioquímica, Facultad de Ciencias, Universidad de Valparaíso, Av. Gran Bretaña 1111, Valparaíso 2360102, Chile; 5 Centro de Investigación Farmacopea Chilena (CIFAR), Universidad de Valparaíso, Santa Marta 183, Valparaíso 2360134, Chile

**Keywords:** fatty acid amide hydrolase, cannabinoid, carboxamide inhibitors, 3D-QSAR, CoMSIA

## Abstract

Fatty Acid Amide Hydrolase (FAAH) is one of the main enzymes responsible for endocannabinoid metabolism. Inhibition of FAAH increases endogenous levels of fatty acid ethanolamides such as anandamide (AEA) and thus consitutes an indirect strategy that can be used to modulate endocannabinoid tone. In the present work, we present a three-dimensional quantitative structure-activity relationships/comparative molecular similarity indices analysis (3D-QSAR/CoMSIA) study on a series of 90 reported irreversible inhibitors of FAAH sharing a piperazine-carboxamide scaffold. The model obtained was extensively validated (q^2^ = 0.734; r^2^ = 0.966; r^2^_m_ = 0.723). Finally, based on the information derived from the contour maps we designed a series of 10 new compounds with high predicted FAAH inhibition (predicted pIC_50_ of the best-proposed compounds = 12.196; 12.416).

## 1. Introduction

The endocannabinoid system (ECS) remains a highly relevant topic in the scientific community as it is involved in several regulatory actions and pathophysiological conditions [1]. Current available knowledge suggests that the ECS is constituted by the cannabinoid receptors, type 1 and 2, the main endogenous ligands anandamide (AEA) and 2-arachidonyl glycerol (2-AG) as well as the enzymes that participate in their biosynthesis (*N*-acyl phosphatidylethanolamine phospholipase D, or NAPE-PLD) and degradation (Fatty Acid Amide Hydrolase or FAAH and Monoacylglycerol Lipase, or MAGL) [2].

FAAH is an integral membrane protein of ~60 kDa (579 amino acids) that belongs to the amidase family of enzymes [3]. It exists as a dimer in its membrane-associated form [3] and is highly expressed in the brain, liver, kidney, and testis. While most other mammalian serine hydrolases possess a Ser-His-Asp catalytic triad, FAAH has a distinctive Ser-Ser-Lys triad [4].

*In vitro* studies show that FAAH can inactivate various lipid amides and allows modifications in both the amide headgroup and lipid acyl chain. In addition to AEA, other bioactive lipid amide substrates include related N-acylethanolamine (NAE), oleamide (cis-9,10-octadecanoamide), and N-acyl taurines (NATs) [4]. 

Endogenous FAAH substrates such as AEA play key regulatory functions in the body and have been implicated in a variety of pathological disorders, including pain, inflammation, anxiety [5], depression and vascular hypertension [6]. Therefore, inhibition of FAAH represents a rational therapeutic approach to treat conditions where higher endocannabinoid activity can be beneficial. Furthermore, as opposed to direct cannabinoid activation, enzyme inhibition offers spatio-temporal control, increasing endocannabinoid activity only at the sites where lipid signaling molecules are being produced. Accordingly, animal studies showed that FAAH inhibitor URB597 elevated endocannabinoid tone without producing motor side effects [7]. For this reason, diverse FAAH inhibitors have been developed [6].

**Figure 1:**
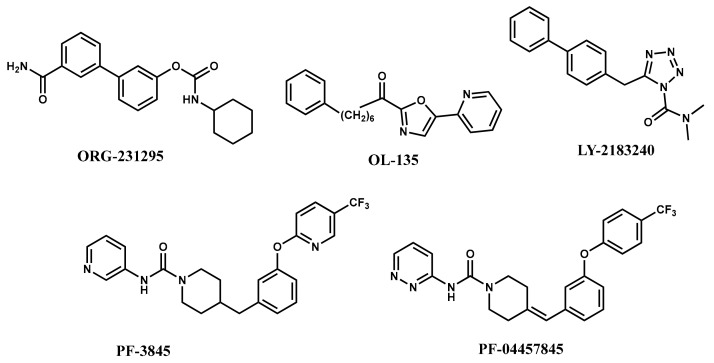
Examples of reported Fatty Acid Amide Hydrolase (FAAH) inhibitors

The first-generation of FAAH inhibitors were designed in order to covalently bind to the catalytic residue Ser241 [3,4]. Despite their ability to block FAAH in *in vitro* pharmacological assays, they remained poor candidates for preclinical studies due to their lack of selectivity [4]. Subsequently, FAAH inhibitors with significantly improved selectivity were developed, including carbamates (ORG-231295), α-ketoheterocycles (OL-135) carbamoyl tetrazoles (LY-2183240), benzothiazole derivatives and piperidine/piperazine ureas [4] (PF-3845, PF-04457845) (Figure 1).

Some quantitative structure-activity relationships (QSAR) studies have been performed on the cannabinoid system (CB1 and CB2 receptors) [8,9,10,11,12,13], however, few structure-activity relationship studies have been performed on FAAH inhibitors, and mostly with carbamate-type structures. Dainese et al. calculated theoretical molecular descriptors in a series of naturally occurring FAAH inhibitors [14]. Käsnänen et al. reported the synthesis and 3D-QSAR studies of carbamate inhibitors [15]. Mor et al. constructed 2D-QSAR equations that could explain the inhibition activity of biphenyl-alkylcarbamates. [6]. Vacondio et al. developed structure-property relationships to explain the hydrolytic stability of carbamate inhibitors [16]. Han et al. reported a comparative molecular field analysis (CoMFA) study on a series of oleoylethanolamide structure inhibitors [17]. To date, there are no 3D-QSAR studies of irreversible inhibitors with the piperazine-carboxamides structure. This type of general structure was shown to have good physical and pharmacokinetic properties and has been reported to be capable of elevating plasma concentrations of AEA, AEP, and AEO in rats [18]. For this reason, the formulation of a QSAR model for the design and prediction of FAAH inhibitor activity based on this structural moiety is significant from a pharmacological point of view.

In the present work, three-dimensional quantitative structure-activity relationships (3D-QSAR) studies based on comparative molecular similarity indices analysis (CoMSIA) were carried out on a set of various reported urea-based FAAH inhibitors. The aim of our 3D-QSAR is to derive useful binding information in order to guide the design of future FAAH inhibitors. The importance of steric, electrostatic and hydrogen-bond characteristics can be analyzed by aligning similar analogues based on key pharmacophoric features [19]. Knowledge of binding requirements can then be used to derive predictive 3D-QSAR models that can, in turn, aid in the design of new inhibitors.

## 2. Results and Discussion

### 2.1. Statistical Results

The statistical results for CoMSIA are presented in Table 1. All possible field combinations were tested for both CoMFA and CoMSIA. In the case of CoMFA, no combination was statistically significant. The CoMSIA models with the highest q^2^ values were those that considered the field combinations SEDA, EDA, EHDA, and SEHDA. The SEDA and EHDA models presented a donor hydrogen-bond contribution of 0.099 and 0.093 respectively. While in the EDA model, the H-bond donor contribution was 0.111 versus 0.889 of the Electrostatic and H-bond Acceptor contributions. The imbalance in the field contribution of these models made us discard them. The final selected model SEHDA, presents a good balance between the field contributions, a high value of q^2^ (0.734) and r^2^_ncv_ (0.937) and higher value of F (138.36). Extensive additional validation was carried out to test the predictive quality of this model.

Table 2 presents a summary of the external validation parameters of the CoMSIA model. The model has a high value of r^2^ (0.966), which indicates satisfactory external predictive power. Nevertheless, as described by Golbraikh and Tropsha, high values of q^2^ and r^2^ (conditions 1 and 2) are not sufficient for model validation. For a reliable predictive capacity, the line of experimental versus predicted activity should be as close as possible to the line *y = x*. This is observed when the conditions (3a or 3b), (4a or 4b) and (5a or 5b) shown in Table 2 are fulfilled. Condition 6 or r^2^_m_ metrics, measures the agreement between the observed and predicted activity. Our derived CoMSIA model satisfied all the conditions for internal and external validation. In addition, we performed the calculation of several external validation descriptors (conditions 7 to 12) [20,21,22]. In all cases, our model passed the validation tests.

Furthermore, the Y-randomization test [23] (Table 3) was applied to assess the robustness of the model (see Table S1 of the Supplementary Material) as previously described by Lorca et al. [24]. The obtained QSAR models show low q^2^ and r^2^_ncv_ values (Table 3).

**Figure 2:**
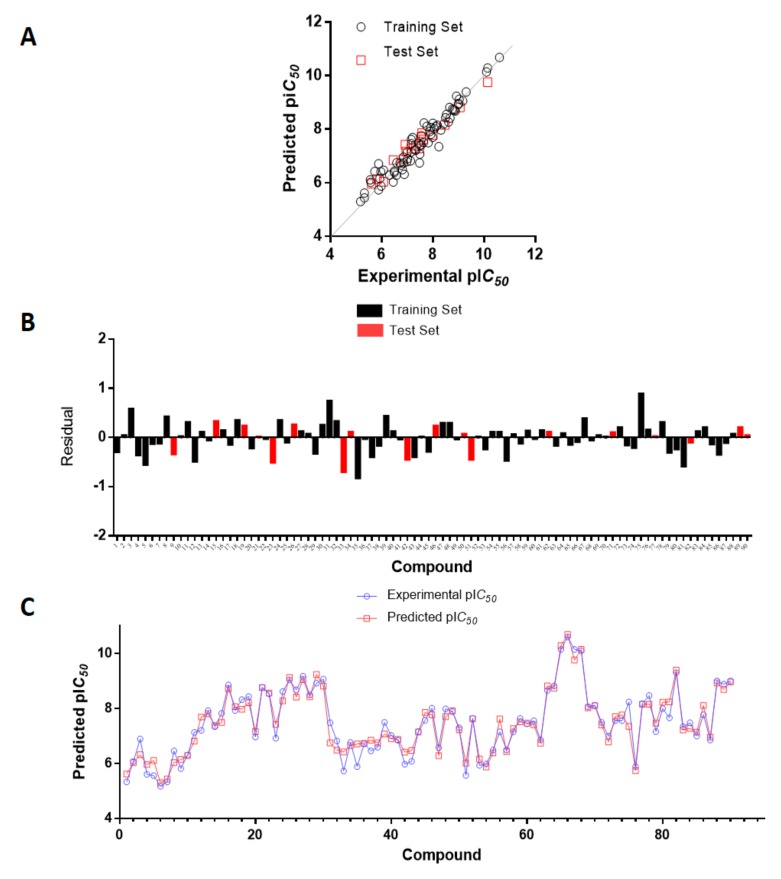
(**A**) Plots of experimental versus predicted pIC_50_ values for the training and test set compounds. (**B**). Residual plots between predicted and experimental values. (**C**) CoMSIA predictions for all molecules in the set.

The values of experimental activity, predicted activity, and the residual values for the best CoMSIA model are shown in Table 4. All the compounds showed low residual values and deviations of the predicted activity greater than a logarithmic unit were not observed. Figure 2A shows a plot of experimental versus predicted activity and the data distribution is close to the *y = x* line. The model shows a good balance in terms of predictive capacity. Forty-two compounds showed negative residual values and 48 presented positive deviations (Figure 2B). The residual range was from −0.82 to 0.89. As shown in Figure 2C the CoMSIA model shows a satisfactory predictive capability throughout the whole data set (training and test set) as well as a good predictive power for both, less active (1, 6 and 7) and most active compounds (65, 66, and 67).

### 2.2. Applicability Domain

In this work we used the method developed by Roy et al. [25] for determination of applicability domain (AD) as previously described by Lorca et al. [24].

The calculation was carried out using the free application available on the author’s page and all compounds were found to be within the domain of applicability, except compounds 83 and 87. These two compounds are the only ones bearing an imidazopyridine or imidazopyrimidine ring and the only difference between them is the position in which the heterocycle is connected to the urea moiety. For this reason, compounds with these heterocyclic systems connected to the urea moiety were not proposed as new molecules.

In summary, the CoMSIA model generated here presents good internal and external validation parameters (q^2^ = 0.734; r^2^ = 0.966), and meets the validation criteria of Tropsha and Roy (r^2^_m_ = 0.723). All the molecules studied are within the applicability domain (except compounds 83 and 87) and the model was validated by the Y-randomization test. Therefore, reliable information can be extracted from the contour maps as discussed in the next section.

### 2.3. Contour Maps Analysis

**Figure 3:**
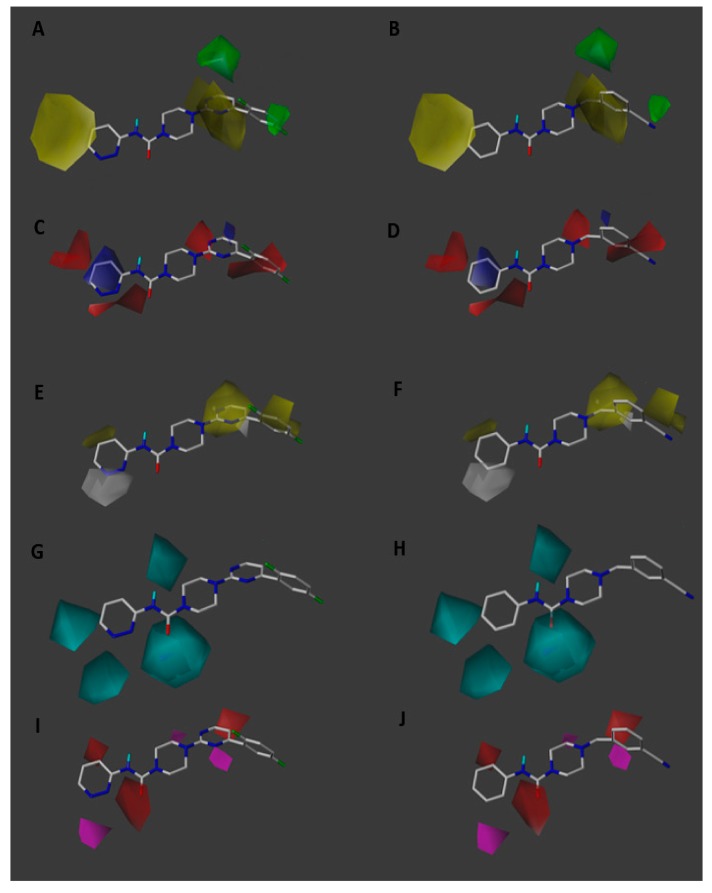
CoMSIA steric (**A,B**), electrostatic (**C,D**), hydrophobic (**E,F**), donor (**G,H**) and acceptor (**I,J**) contour maps around compounds 66 (left) and 6 (right), the most active and least active of the series respectively. Sterically favored areas are in green and disfavored areas are in yellow. Electropositive favoured areas are in blue and electronegative favoured areas are in red. Hydrophobic favoured areas are in yellow and unfavourable areas in grey. Donor and acceptor favoured areas are in cyan and magenta respectively, and donor and acceptor unfavourable areas are in purple and red, respectively.

The result of a 3D-QSAR study can be visualized graphically unlike a traditional 2D-QSAR equation. Contour maps represented by colored polyhedrons can be seen around the molecules. The maps obtained in our study correspond to the steric, electrostatic, hydrophobic, H-bond donor and H-bond acceptor contour maps. Regions where a molecular property is favorable or unfavorable are indicated by different colored polyhedrons. Figure 3 presents the different maps around the most active compound (66, pIC_50_ = 10.602; on the left) and the least active compound (6, pIC_50_ = 5.176; on the right). 

#### 2.3.1. Steric Contour Map

The steric contour map shows a large yellow polyhedron close to the pyridazine ring of the most active compound indicating that bulky substituents in this region should be avoided in order to favor biological activity (Figure 3A,B). Alternatively, a smaller five-membered ring could replace the pyridazine ring following the same steric requirement. This can be seen in the proposed molecules (Table 5) where the analog 2x shows a considerable increase in predicted activity when the pyridazine ring is replaced by a pyrazole ring. This relation is further supported by compounds 54 and 55 (Table 6) which bear phenyl substituents in the corresponding position and show low activity consistent with their bulkier nature. 

On the other side, the yellow polyhedron that surrounds the pyrimidine ring indicates that reducing the size of this ring or replacing it with a smaller linker while maintaining the electronic properties would be beneficial for activity. Additionally, the green polyhedrons around the ortho and meta positions of the disubstituted benzene ring indicate that bulky substituents in these positions can be favorable for activity. This can also be seen in Table 5 where substitution with a methyl group considerably increases the predicted activity in all the proposed molecules. For the least active compounds (Figure 3B), the steric factor by itself does not seem to explain the lower activity values.

#### 2.3.2. Electrostatic Contour Map

Regarding the electrostatic contour maps, the red polyhedrons around the pyridazine ring (Figure 3C) highlight the importance of nitrogenated heterocycles that can confer electronegative areas in this region. This may explain why molecules 1, 6 and 7 (Table 6) bearing benzene rings with more homogeneous charge distribution show lower activity. The blue polyhedron inside the pyridazine ring shows that an electropositive center is beneficial for activity, therefore, incorporating electro withdrawing substituents in positions 5 and 6 of the pyridazine ring could increase activity. This electron distribution with an electron rich edge and electron deficient center suggests possible pi-stacking or pi-cation interactions with the target enzyme. Similarly, the expansion of the blue contour at position 4 of the pyridazine ring indicates that electron withdrawing substituents particularly at this position are favorable for activity. In agreement with this, proposed analogues 1x, 9x and 10x (Table 5) that follow this substitution pattern display high predicted activity. The polarization of the carbon atom directly attached to the electroattractive or electronegative groups nitrile and fluorine lower the electron density right where the blue polyhedron lies. 

On the other side, the red contour over the pyrimidinic nitrogen (Figure 3C) shows the importance of an electronegative atom at this position as is present in the most active molecules (65, 66, 67 and 68 from Table 6) and absent in the least active one (compound 6). Likewise, an electron rich benzene ring is favorable and thus replacing the fluorine atom for an electrodonating group would be recommended. Accordingly, the proposed molecules 9x and 10x substituted with electrodonating methylene groups show the highest predicted activities. In Figure 3D the red contour over the electron deficient nitrile carbon atom may explain the lower activity of this analog.

#### 2.3.3. Hydrophobic Contour Map

The hydrophobic contour maps (Figure 3E,F) show a gray polyhedron over the pyridazine nitrogen atoms of the most active molecules, indicating that incorporating hydrophilic atoms in this region can favor activity. The yellow polyhedron over the pyrimidine ring shows that a hydrophobic linker region is important. Additional yellow polyhedrons surrounding the disubstituted benzene ring suggest hydrophobic substituents in the meta and ortho positions can also increase activity. Following both the hydrophobic and the previously mentioned steric requirement all proposed molecules (Table 5) possess an ortho-methyl substituent in the benzene ring.

#### 2.3.4. Donor and Acceptor Contour Maps

In the hydrogen bond donor map (Figure 3G,H) cyan polyhedrons surrounding the pyridazine ring suggest that incorporation of hydrogen bond donor groups can favor activity. For this reason, the proposed molecule 3x (Table 5) was designed with a hydroxyl group able to form hydrogen bonds. Furthermore, cyan polyhedrons around the urea linker suggest that the urea moiety is involved in a hydrogen bond interaction with the target enzyme.

Finally, the hydrogen bond acceptor map shows red polyhedrons next to position 4 of the pyridazine ring (Figure 3I,J), position 5 of the pyrimidine ring and over the urea carbonyl. Indicating that hydrogen bond acceptor groups in these positions is unfavorable for activity. Therefore, using different linker groups without hydrogen bond acceptor groups could be advisable in order to design new inhibitors. 

### 2.4. Design of New FAAH Inhibitors

Based on the information obtained from the contour maps, we have designed a series of compounds evaluating multiple combinations of fragments. Substituents and functional groups were proposed, taking into consideration the electronic, steric, hydrophobic and hydrogen bonding properties suggested by the contour maps. Table 5 shows the compounds that presented the best predicted inhibitory activity. All proposed molecules have better predicted activity than the most active compound in the series (66, pIC_50_ = 10.602). In general, the presence of 6-member rings or fused systems on the left side did not greatly increase activity (1x pIC_50_ = 10.889; 4x pIC_50_ = 10.990). However, the insertion of a pyrazole ring generated derivatives with a significant increase in the pIC_50_ value (best compounds: 9x, pIC_50_ = 12.416; 10x, pIC_50_ = 12.196). This is because the pyrazole system meets the electronic, hydrophilic and hydrogen bonding requirements suggested by the contour maps. On the right side, the insertion of halogens and alkyl groups slightly increased the activity. 

Due to the reported potential toxicity of heterocyclic compounds similar to those presented in Table 5 [26], we conducted a predictive toxicity study using the PreAdmet online platform (https://preadmet.bmdrc.kr/adme/). The calculation showed that only compound 1x presents a high risk of toxicity mediated by affinity to the anti-target hERG.

## 3. Materials and Methods

### 3.1. Molecular Alignment

**Figure 4:**
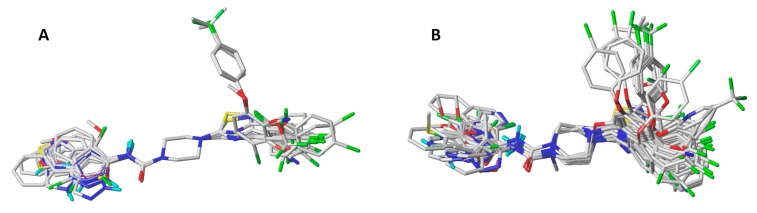
The superimposed structures of all compounds used in the CoMSIA model. (**A**) Atom fit method. (**B**) Distill rigid method.

CoMSIA studies were performed with Sybyl X-1.2 software (1.2, Tripos International, St. Louis, MS, USA) [27] installed in a Windows 10 environment on a PC with an Intel core i7 CPU. Each compound was drawn in ChemDraw and then geometry optimized using MM2 molecular mechanics as implemented in ChemBio3D software (15.1.0, PerkinElmer, Waltham, MA, USA). The structures were further minimized by Tripos force field implemented in Sybyl. MMFF94 charges were assigned to each atom. Among the techniques to perform molecular alignment are: (1) atom-by-atom alignments using a common fragment, (2) rigid alignment that minimizes rms distance, (3) flexible alignments [28] and (4) receptor guided alignments. In the present study, the first two options were carried out (Figure 4). In the first case, the piperazinyl-urea nucleus was chosen as the common scaffold for alignment. In the second case, the alignment was carried out using the Distill rigid protocol, as implemented in Sybyl. The best results in terms of statistical validation (*q^2^*, *N*) were for the atom-by-atom alignment, which was chosen as the basis for the development of the models.

### 3.2. CoMSIA Field Calculation

To derive the CoMSIA descriptor fields, the aligned training set molecules were placed in a 3D cubic lattice with grid spacing of 2Å in x, y, and z directions such that the entire set was included in it. The CoMSIA analysis, the standard settings (probe with charge +1.0, radius 1Å, hydrophobicity +1.0, hydrogen-bond donating +1.0, hydrogen bond accepting +1.0 [29]) were used to calculate five different fields: steric, electrostatic, hydrophobic, donor and acceptor. Gaussian-type distance dependence was used to measure the relative attenuation of the field position of each atom in the lattice, and led to much smoother sampling of the fields around the molecules when compared to CoMFA. The default value of 0.3 was set for the attenuation factor α.

### 3.3. Data Set Selection and Inhibitory Activity

**Figure 5:**
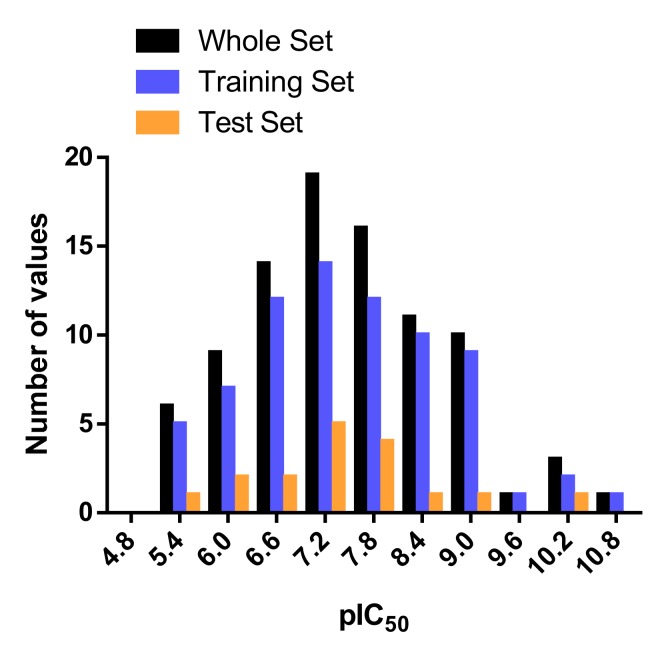
Histogram of frequency distribution data.

CoMSIA studies were performed on a set of 90 piperazinyl urea derivatives reported in the literature [18,30,31,32,33,34,35,36] (Table 6). The derivatives displayed potent fatty acid amide hydrolase (FAAH) inhibitors activity. The IC_50_ values were converted to pIC_50_ (–logIC_50_). The compounds were divided into training (73 compounds, 81%) and test sets (17 compounds, 19%), ensuring that both sets contained structurally diverse compounds with high, medium and low activity, and a uniform distribution to avoid possible problems during the external validation. The distribution of pIC_50_ values for the whole set, the training set and the test set is shown in Figure 5. In all three cases the biological activity follows a Gaussian distribution.

### 3.4. Internal Validation and Partial Least Squares (PLS) Analysis 

PLS analysis [37] was used to construct a linear correlation between the CoMFA and CoMSIA descriptors (independent variables) and the activity values (dependent variables) as previously described by Lorca et al. [24].

### 3.5. External Validation

The models were subjected to external validation criteria according to the proposed test by Golbraikh and Tropsha [38,39], which considers a QSAR model predictive, if the following conditions are satisfied:

(1) $$q^{2}>0.5$$

(2) $$r^{2}>0.6$$

(3) $$\frac{\left(r^{2}-r_{0}^{2}\right)}{r^{2}}<0.1\;\mathrm{or}\;\frac{\left(r^{2}-r_{\;0}^{\prime 2}\right)}{r^{2}}<0.1$$

(4) $$0.85\leq 1.15\;\mathrm{or}\;0.85\leq k^{\prime }\leq 1.15$$

It has been demonstrated [38] that to adequately assess the predictive ability of a QSAR model the above criteria are necessary. 

Furthermore, the external predictive power of the developed 3D-QSAR models using the test set was examined by considering *r^2^_m_* metrics as shown below [40]:

(5) $$r_{m}^{2}=r^{2}\;\left(1-\left|\sqrt{r^{2}-r_{0}^{2}}\right|\right)$$

where *r^2^* and *r^2^_0_* are squared correlation coefficients between the observed and predicted activities of the test set with and without intercept, respectively. For a significant external model validation, the value of *r^2^_m_* should be greater than 0.5.

Additionally, the following descriptors were calculated:

(6) $$Q_{F1}^{2}=1-\;\frac{\sum_{i=1}^{n_{EXT}}{\left(y_{i}-{\hat{y}}_{i}\right)}^{2}}{\sum_{i=1}^{n_{EXT}}{\left(y_{i}-{\bar{y}}_{TR}\right)}^{2}}$$

(7) $$Q_{F2}^{2}=1-\;\frac{\sum_{i=1}^{n_{EXT}}{\left(y_{i}-{\hat{y}}_{i}\right)}^{2}}{\sum_{i=1}^{n_{EXT}}{\left(y_{i}-{\bar{y}}_{EXT}\right)}^{2}}$$

(8) $$Q_{F3}^{2}=1-\;\frac{\sum_{i=1}^{n_{EXT}}{\left(y_{i}-{\hat{y}}_{i}\right)}^{2}/n_{EXT}}{\sum_{i=1}^{n_{EXT}}{\left(y_{i}-{\bar{y}}_{TR}\right)}^{2}/n_{TR}}$$

(9) $$CCC=\frac{2\sum_{i=1}^{n_{EXT}}\left(y_{i}-\bar{y}\right)\left({\hat{y}}_{i}-\bar{\hat{y}}\right)}{\sum_{i=1}^{n_{EXT}}{\left(y_{i}-\bar{y}\right)}^{2}+\;\sum_{i=1}^{n_{EXT}}{\left({\hat{y}}_{i\;}-\bar{\hat{y}}\right)}^{2}+\;n_{EXT}{\left(\bar{y}-\bar{\hat{y}}\right)}^{2}}$$

(10) $$\Delta r_{m}^{2}=\left|r_{m}^{2}-r_{m}^{\prime 2}\right|$$

where, TR = training set, EXT = external prediction set, $y_{i}$ = experimental data values, ${\hat{y}}_{i}$ = predicted data values, $\bar{y}$ = average of the experimental data values, $\bar{\hat{y}}$ = average of the predicted data values. Finally, $r_{m}^{2}$ is calculated using the experimental values on the ordinate axis, while $r_{m}^{\prime 2}$ using them on the abscissa.

### 3.6. Applicability Domain (AD) Calculation

The AD was evaluated based on the simple standardization method reported by Roy et al. [25] and as described by Lorca et al. [24]. 

## 4. Conclusions

**Figure 6:**
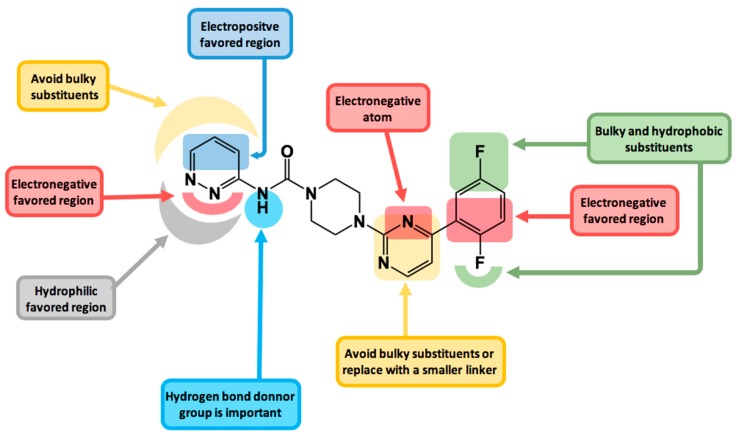
Main structure-activity relationships derived from this study.

In this contribution, a 3D-QSAR CoMSIA study was carried out on an extensive database of 90 irreversible inhibitors of the enzyme FAAH with a pyrimidinyl-piperazine-carboxamide general structure. The best model obtained considered all the field contributions, being the electrostatic and hydrogen-bond acceptor properties the ones that contributed most to the activity (30.4% and 33.0% respectively). The model was validated internally (q^2^ = 0.734) and externally (r^2^_test_ = 0.966) and was also submitted to Tropsha validation criteria, r^2^_m_ calculation (0.723) and Y-randomization test, passing all tests. The information derived from the contour maps was used to design a series of new compounds that showed promising predicted activities (pIC_50_ of the most active compounds = 12.196 and 12.416). The main structure-activity relationships found in this study and summarized in Figure 6 are a useful tool to guide the future design of new FAAH inhibitors. The extensive database used in this study could motivate future work complementing the information obtained from contour maps with QSAR studies by Dragon-based descriptors [41].

## Figures and Tables

**Table 1:** Statistical parameters and field combinations for comparative molecular similarity indices analysis (CoMSIA).

Model	q^2^	N	SEP	SEE	r^2^_ncv_	F	Field Contributions				
							S	E	H	D	A
CoMSIA-S	0.293	3	1.042	0.903	0.470	20.383	1				
CoMSIA-E	0.534	10	0.893	0.476	0.867	40.592		1			
CoMSIA-H	0.317	4	1.032	0.788	0.602	25.722			1		
CoMSIA-D	0.253	8	1.112	1.031	0.359	4.482				1	
CoMSIA-A	0.520	5	0.871	0.638	0.743	38.785					1
CoMSIA-SE	0.519	10	0.907	0.417	0.898	54.799	0.314	0.686			
CoMSIA-SEH	0.534	8	0.879	0.216	0.977	110.701	0.183	0.492	0.324		
CoMSIA-SEHD	0.628	7	0.779	0.382	0.910	94.448	0.159	0.458	0.260	0.123	
CoMSIA-SEHA	0.688	7	0.713	0.332	0.933	128.486	0.131	0.346	0.177		0.347
CoMSIA-SED	0.626	9	0.793	0.382	0.913	73.600	0.245	0.639		0.116	
CoMSIA-SEA	0.725	7	0.670	0.350	0.925	114.665	0.182	0.387			0.421
CoMSIA-SEDA	0.765	7	0.620	0.327	0.934	132.475	0.154	0.357		0.099	0.389
CoMSIA-SH	0.316	4	1.033	0.772	0.618	27.502	0.408		0.592		
CoMSIA-SD	0.364	19	1.128	0.525	0.862	17.433	0.814			0.186	
CoMSIA-SA	0.572	7	0.836	0.484	0.857	55.453	0.344				0.656
CoMSIA-SHD	0.426	3	0.939	0.799	0.585	32.396	0.219		0.479	0.303	
CoMSIA-SHA	0.529	6	0.870	0.483	0.855	64.909	0.201		0.303		0.496
CoMSIA-SDA	0.719	7	0.678	0.404	0.900	83.485	0.235			0.210	0.555
CoMSIA-SHDA	0.673	7	0.731	0.366	0.918	103.744	0.156		0.240	0.164	0.440
CoMSIA-EH	0.550	10	0.877	0.391	0.911	63.307		0.537	0.427		
CoMSIA-ED	0.616	9	0.804	0.407	0.902	64.163		0.856		0.144	
CoMSIA-EA	0.701	6	0.693	0.408	0.896	95.020		0.498			0.502
CoMSIA-EHD	0.641	8	0.771	0.376	0.915	85.695		0.525	0.343	0.132	
CoMSIA-EHA	0.691	7	0.710	0.390	0.925	115.138		0.390	0.234		0.376
CoMSIA-EDA	0.752	7	0.636	0.366	0.918	103.539		0.453		0.111	0.437
CoMSIA-EHDA	0.742	8	0.654	0.311	0.942	128.936		0.341	0.211	0.093	0.355
CoMSIA-HD	0.428	9	0.981	0.528	0.834	35.186			0.804	0.196	
CoMSIA-HA	0.537	6	0.862	0.493	0.849	61.685			0.426		0.574
CoMSIA-HDA	0.682	10	0.738	0.331	0.936	90.828			0.356	0.155	0.490
CoMSIA-DA	0.705	11	0.716	0.465	0.876	39.132				0.240	0.760
CoMSIA-ALL	0.734	7	0.659	0.320	0.937	138.360	0.110	0.304	0.156	0.100	0.330

q^2^ = the square of the LOO cross-validation (CV) coefficient; N = the optimum number of components; SEP = standard error of prediction; SEE is the standard error of estimation of non CV analysis; r^2^_ncv_ is the square of the non CV coefficient; F is the F-test value; S, E, H, D and A are the steric, electrostatic, hydrophobic, hydrogen-bond donor, and hydrogen-bond acceptor contributions respectively. No statistically significant models were found for CoMFA.

**Table 2:** Summary of external validation parameters for CoMSIA.

Condition	Parameters	Threshold Value	CoMSIA
1	*q^2^*	>0.5	0.734
2	*r^2^*	>0.6	0.966
3a	*r_0_^2^*	Close to value of *r^2^*	0.920
3b	*r^′^_0_^2^*	Close to value of *r^2^*	0.944
4a	*k*	0.85 < *k* < 1.15	1.004
4b	*k^′^*	0.85 < k′ < 1.15	0.995
5a	*(r^2^−r^2^_0_)/r^2^*	<0.1	0.048
5b	*(r^2^−r’^2^_0_)/r^2^*	<0.1	0.023
06	|*r^2^_0_−r′^2^_0_*|	<0.3	0.024
7	$r_{m}^{2}$	>0.5	0.723
8	*Q^2^_F1_*	>0.7	0.944
9	*Q^2^_F2_*	>0.7	0.943
10	*Q^2^_F3_*	>0.7	0.951
11	CCC	>0.85	0.967
12	∆r^2^_m_	<0.2	0.056

q^2^ = the square of the LOO cross-validation (CV) coefficient; r^2^ is the regression coefficient for the test set exclusively; r_0_^2^ and k are the correlation coefficient between the actual and predicted activities for test set and the respective slope of regression; and r_0_’^2^ and k’ are the correlation coefficient between the predicted and actual activities for test set and the respective slope of regression. r^2^_m_ was defined in equation 5. Parameters 8–12 are defined in the methods section.

**Table 3:** Y-randomization test for CoMSIA model.

Iteration	q^2^	r^2^_ncv_	Iteration	q^2^	r^2^_ncv_
Random 1	−0.013	0.107	Random 6	0.006	0.119
Random 2	−0.030	0.087	Random 7	−0.093	0.183
Random 3	−0.052	0.082	Random 8	0.085	0.188
Random 4	−0.198	0.108	Random 9	−0.034	0.086
Random 5	−0.202	0.179	Random 10	−0.100	0.073

**Table 4:** Experimental and predicted pIC_50_ and residual values for the analyzed compounds obtained with the CoMSIA model.

Mol	Exp. pIC_50_	Pred. pIC_50_	Residual	Mol	Exp. pIC_50_	Pred. pIC_50_	Residual
**1**	5.331	5.620	−0.29	**46 ^t^**	8.009	7.774	0.23
**2 ^t^**	6.076	6.034	0.04	**47**	6.575	6.286	0.29
**3**	6.893	6.317	0.58	**48**	7.987	7.699	0.29
**4 ^t^**	5.607	5.961	−0.35	**49**	7.886	7.915	−0.03
**5**	5.558	6.107	−0.55	**50**	7.301	7.231	0.07
**6**	5.176	5.295	−0.12	**51**	5.574	6.018	−0.44
**7**	5.331	5.438	−0.11	**52 ^t^**	7.638	7.624	0.01
**8**	6.456	6.032	0.42	**53**	5.933	6.164	−0.23
**9 ^t^**	5.815	6.141	−0.33	**54**	5.984	5.876	0.11
**10**	6.310	6.291	0.02	**55**	6.495	6.381	0.11
**11**	7.131	6.816	0.31	**56**	7.155	7.619	−0.46
**12**	7.208	7.689	−0.48	**57**	6.495	6.436	0.06
**13**	7.921	7.810	0.11	**58 ^t^**	7.155	7.268	−0.11
**14 ^t^**	7.337	7.386	−0.05	**59**	7.638	7.512	0.13
**15**	7.824	7.497	0.33	**60**	7.444	7.467	−0.02
**16**	8.854	8.718	0.14	**61**	7.553	7.408	0.14
**17**	7.921	8.062	−0.14	**62**	6.854	6.741	0.11
**18**	8.319	7.972	0.35	**63**	8.658	8.820	−0.16
**19**	8.432	8.206	0.23	**64**	8.824	8.740	0.08
**20 ^t^**	6.959	7.170	−0.21	**65**	10.143	10.287	−0.14
**21**	8.770	8.758	0.01	**66**	10.602	10.685	−0.08
**22**	8.538	8.559	−0.02	**67 ^t^**	10.143	9.759	0.38
**23 ^t^**	6.921	7.421	−0.50	**68**	10.097	10.144	−0.05
**24**	8.620	8.273	0.35	**69**	8.061	8.023	0.04
**25**	9.036	9.126	−0.09	**70**	8.114	8.106	0.01
**26**	8.678	8.415	0.26	**71**	7.495	7.397	0.10
**27**	9.174	9.057	0.12	**72**	6.987	6.787	0.20
**28**	8.495	8.427	0.07	**73 ^t^**	7.553	7.707	−0.15
**29**	8.921	9.236	−0.32	**74**	7.553	7.757	−0.20
**30 ^t^**	9.066	8.814	0.25	**75**	8.237	7.347	0.89
**31**	7.482	6.743	0.74	**76**	5.886	5.739	0.15
**32**	6.818	6.490	0.33	**77**	8.174	8.153	0.02
**33**	5.731	6.422	−0.69	**78 ^t^**	8.469	8.155	0.31
**34**	6.762	6.654	0.11	**79**	7.161	7.463	−0.30
**35**	5.886	6.710	−0.82	**80**	8.000	8.225	−0.23
**36**	6.714	6.734	−0.02	**81**	7.658	8.237	−0.58
**37 ^t^**	6.460	6.848	−0.39	**82**	9.301	9.394	−0.09
**38**	6.590	6.753	−0.16	**83**	7.337	7.216	0.12
**39**	7.499	7.072	0.43	**84 ^t^**	7.482	7.278	0.20
**40**	7.018	6.901	0.12	**85**	7.000	7.134	−0.13
**41 ^t^**	6.845	6.878	−0.03	**86**	7.770	8.110	−0.34
**42**	5.972	6.412	−0.44	**87**	6.854	6.957	−0.10
**43**	6.079	6.471	−0.39	**88**	9.000	8.926	0.07
**44**	7.161	7.148	0.01	**89**	8.886	8.686	0.20
**45 ^t^**	7.574	7.855	−0.28	**90**	9.000	8.960	0.04

^t^ test set compound.

**Table 5:** The proposed structures of new molecules and their predicted pIC_50_ values using the CoMSIA model.

N°	Structure	Pred. pIC_50_	N°	Structure	Pred. pIC_50_
**1x**	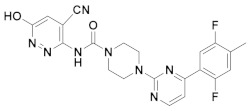	10.889	**6x**	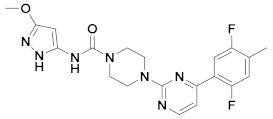	11.744
**2x**	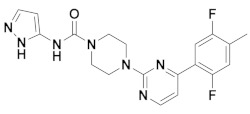	11.388	**7x**	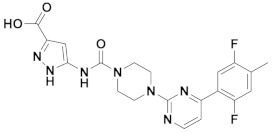	11.599
**3x**	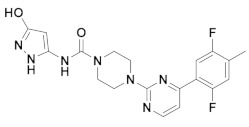	11.490	**8x**	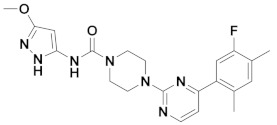	11.822
**4x**	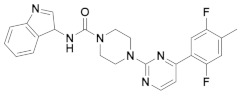	10.990	**9x**	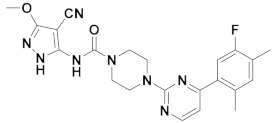	12.416
**5x**	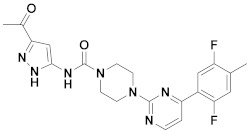	11.253	**10x**	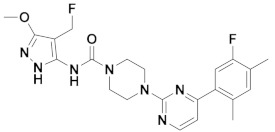	12.196

**Table 6:** Chemical structure, IC_50_ (nM) and pIC_50_ values of the studied molecules.

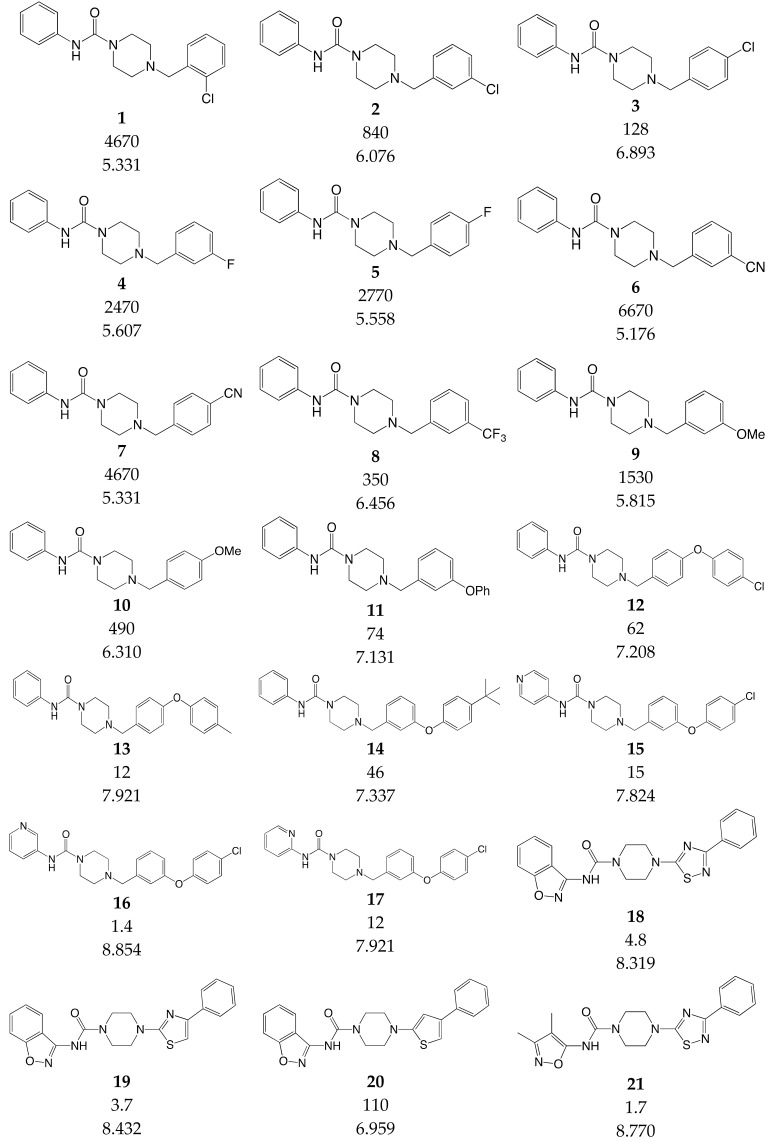
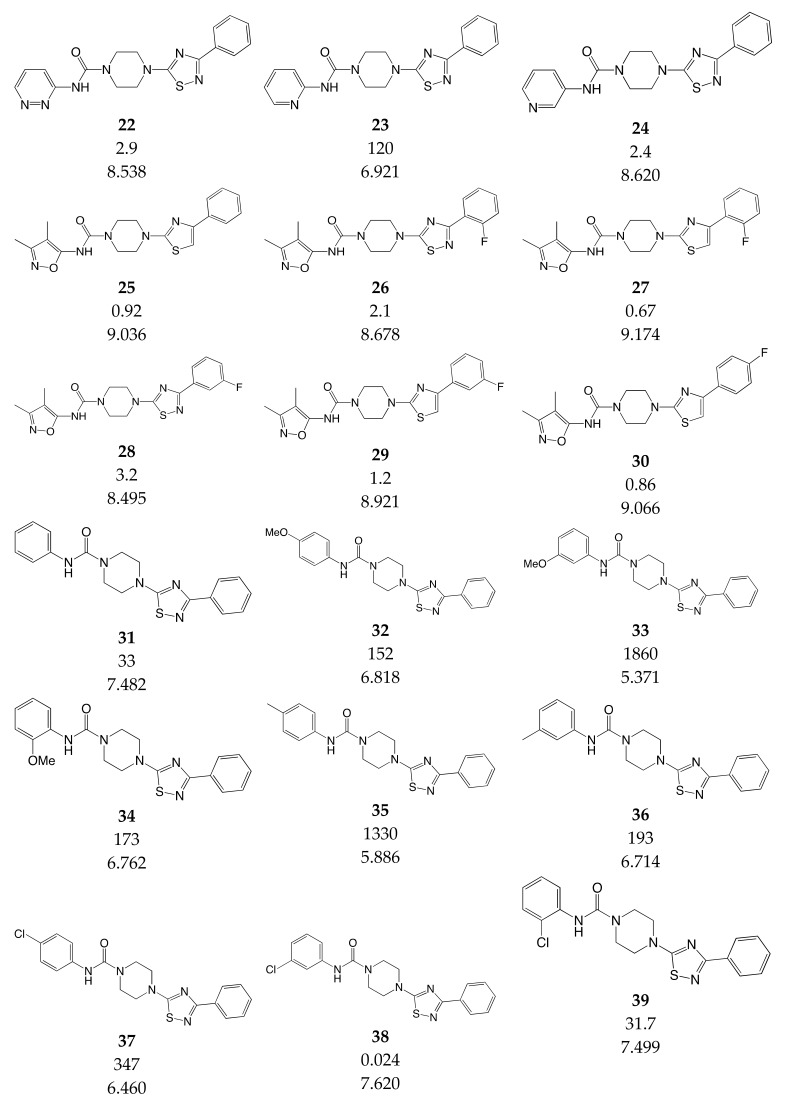
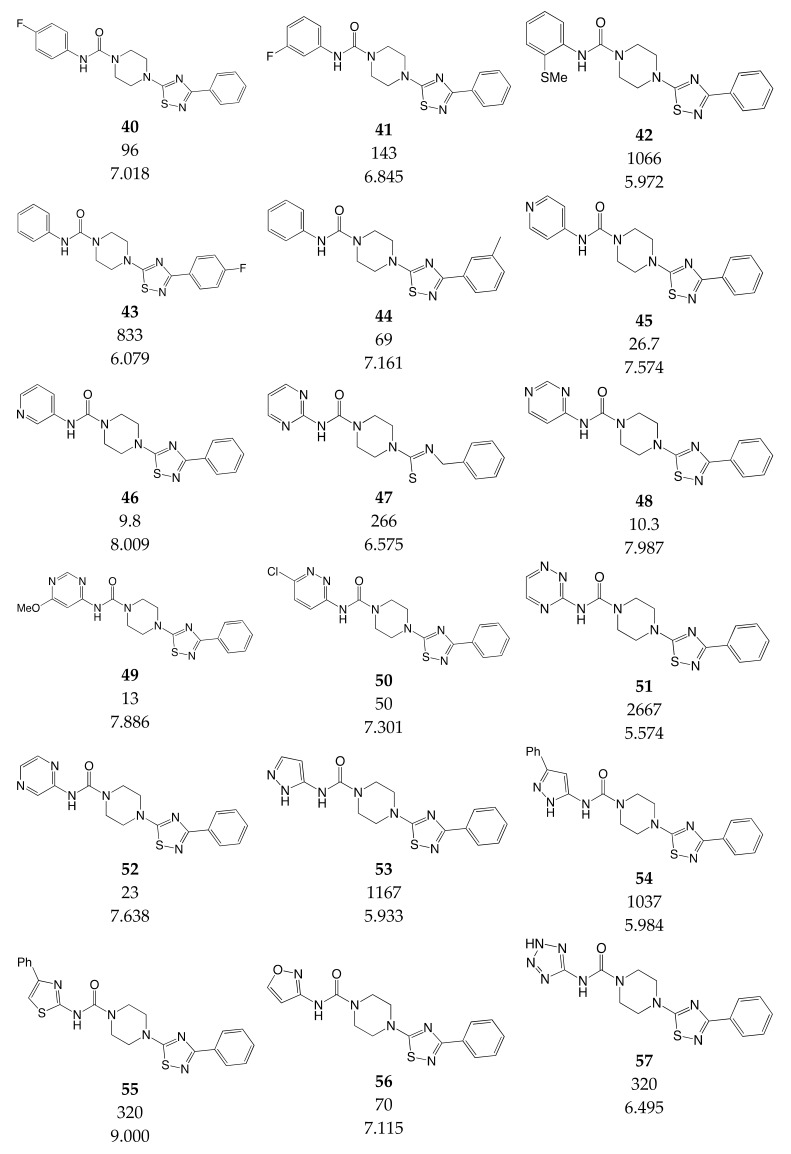
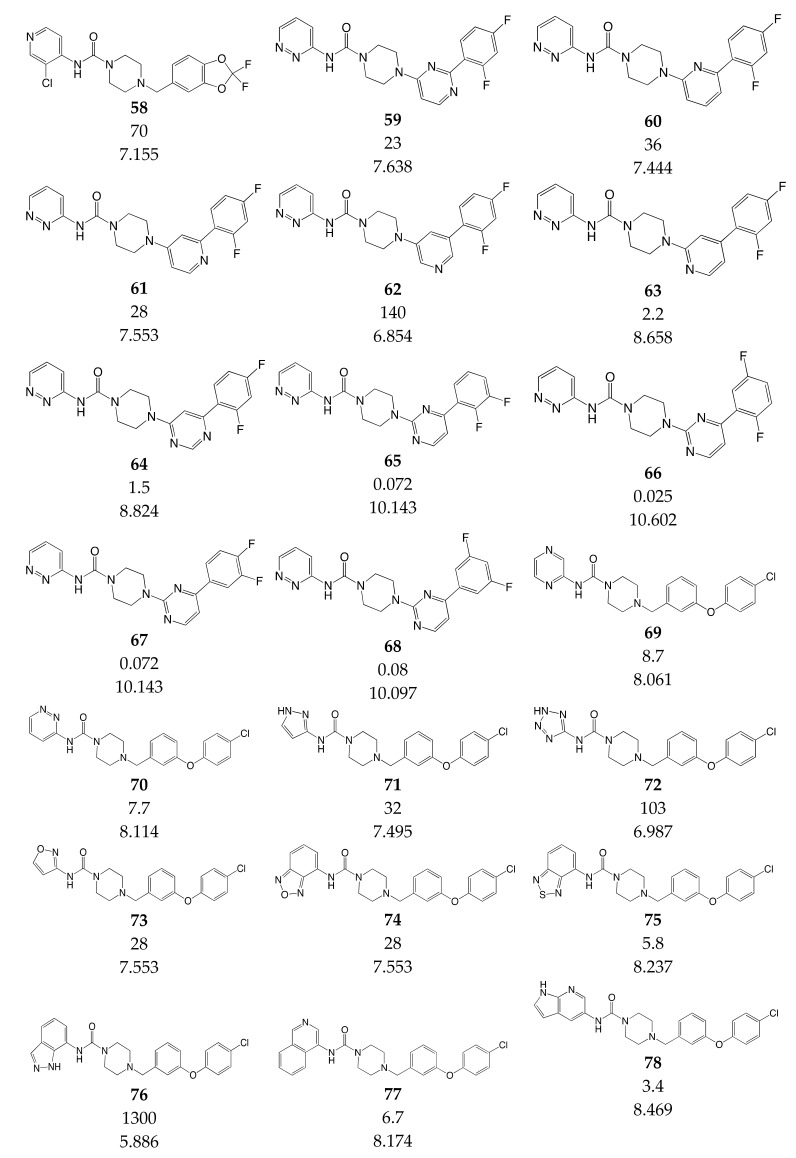
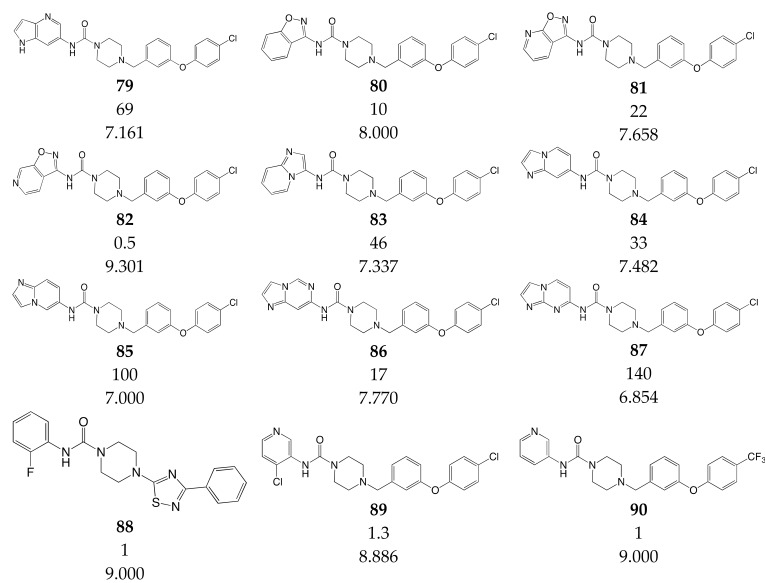

IC_50_ = Half maximal inhibitory concentration; pIC_50_ = −logIC_50_; M = mol·L^−1^

## Supplementary Materials

Supplementary materials can be found at https://www.mdpi.com/1422-0067/20/10/2510/s1.

## Abbreviations

3D-QSAR	Three-dimensional Quantitative Structure-Activity Relationship
CoMSIA	Comparative Molecular Similarity Indices Analysis
FAAH	Fatty Acid Amide Hydrolase
CB	Cannabinoid
