# Supplementary material for: Three-Dimensional Quantitative Structure-Activity Relationships (3D-QSAR) on a Series of Piperazine-Carboxamides Fatty Acid Amide Hydrolase (FAAH) Inhibitors as a Useful Tool for the Design of New Cannabinoid Ligands
Source: Int J Mol Sci. 2019 May 21;20(10):2510. doi: 10.3390/ijms20102510 (PMC6566251; doi:10.3390/ijms20102510)
Supplement: Supplementary file 1 [file ijms-20-02510-s001.pdf]

Table S1. Randomizations of biological activity for Y-randomizarion test.

Random 1	Random 2	Random 3	Random 4	Random 5	Random 6	Random 7	Random 8	Random 9	Random 10
9.036	7.886	7.000	6.590	5.886	7.921	6.310	7.301	7.824	5.984
8.174	7.553	5.984	8.114	7.444	9.301	8.000	7.553	7.301	6.854
7.208	6.854	7.208	5.972	6.893	8.061	6.456	8.658	8.114	7.018
5.558	5.731	6.762	7.444	6.854	7.161	8.061	10.097	5.574	5.731
7.921	7.301	5.886	7.824	7.161	8.886	9.000	5.984	5.886	7.495
7.553	8.114	5.731	7.921	5.933	7.301	7.638	8.495	7.161	6.310
6.818	5.933	6.987	8.620	6.987	8.824	7.301	9.174	5.176	6.575
9.000	7.495	6.854	6.854	6.310	7.018	8.174	9.000	6.854	9.000
8.061	7.000	7.886	10.097	7.921	7.131	8.678	9.000	8.000	8.319
7.155	5.972	7.018	6.714	7.161	7.987	8.538	6.762	8.620	8.495
6.987	9.000	5.331	8.824	7.638	7.921	5.886	6.079	9.301	8.921
6.079	7.921	5.574	6.762	10.143	8.770	7.553	7.886	8.061	10.097
5.984	6.495	10.602	8.495	8.854	6.495	9.000	8.174	9.174	9.036
7.553	7.638	8.319	10.143	10.602	8.678	10.143	8.678	8.658	5.176
8.658	6.987	9.000	9.301	10.097	6.818	8.658	7.658	7.553	7.301
7.301	6.893	8.678	7.770	8.921	9.000	10.097	7.131	6.310	5.886
5.731	6.762	9.036	7.638	8.620	7.495	6.575	5.886	7.638	7.000
5.886	9.301	9.301	7.495	7.301	6.762	8.854	7.499	6.714	8.538
5.972	5.331	7.824	7.301	8.538	8.114	7.553	5.176	5.331	5.558
7.161	9.174	5.933	5.176	5.886	5.331	8.319	7.553	8.921	6.987
6.310	8.538	8.770	5.574	8.114	8.319	5.731	6.456	6.590	10.602
6.893	7.208	7.155	9.000	8.174	8.237	7.824	5.972	7.444	7.824
8.678	7.987	8.061	7.987	6.818	6.495	8.824	5.886	7.155	8.886
5.574	7.824	7.131	7.337	6.079	10.602	5.331	8.237	7.658	6.495
8.620	8.921	8.886	7.553	7.000	10.097	6.495	7.921	6.893	6.079
6.854	7.337	7.987	7.553	7.824	7.337	5.972	5.574	8.678	7.658
6.575	9.000	7.770	10.602	5.984	7.658	8.495	8.854	7.553	5.331
8.538	10.097	6.575	5.331	7.987	7.553	6.818	6.987	6.854	8.678
7.000	10.602	7.658	9.036	8.495	6.590	7.000	7.208	6.456	6.893
7.499	6.590	6.456	6.079	7.553	6.714	6.762	7.921	6.762	8.620
10.097	8.319	9.174	7.208	9.036	10.143	7.482	7.444	7.482	8.658
7.161	10.143	7.638	7.131	7.018	9.174	8.620	7.482	5.558	5.933
8.921	8.886	10.143	5.886	7.921	9.000	5.984	7.161	7.131	6.714
5.331	8.061	7.499	6.495	7.770	6.079	8.237	7.638	10.602	6.818
5.886	7.161	6.590	7.161	6.854	6.987	5.886	7.987	6.079	7.921
5.331	9.036	6.495	7.000	9.174	7.000	7.987	5.731	9.000	9.000
6.762	7.444	8.495	8.000	9.000	8.000	5.176	6.575	8.854	9.301
8.237	6.495	8.174	8.921	6.590	6.854	6.854	8.114	7.921	8.061
7.444	8.824	8.432	8.658	5.972	7.886	7.499	6.310	6.495	8.114
8.770	5.558	6.310	8.886	6.495	6.310	5.933	7.770	6.495	6.456
8.114	8.770	8.114	9.174	6.456	8.854	7.131	5.331	7.000	7.131

7.131	5.331	6.079	6.987	7.155	5.933	8.432	6.495	8.824	8.237
7.337	6.310	7.921	5.984	5.558	8.658	7.886	7.000	5.331	8.000
7.018	5.176	8.620	7.658	5.331	6.893	7.161	8.824	7.987	8.174
6.495	6.575	5.176	6.575	9.000	8.620	6.854	8.432	5.972	5.972
6.854	7.155	5.886	7.886	6.575	6.456	6.495	7.155	10.097	6.495
10.143	6.079	6.893	8.770	7.337	7.824	7.155	7.337	5.984	6.854
8.000	8.432	9.000	7.018	5.176	5.984	7.018	6.495	7.495	7.208
8.319	7.921	7.553	7.482	6.495	8.495	5.574	5.331	8.237	7.553
7.658	5.886	8.854	5.331	8.432	7.444	9.174	6.893	8.319	5.574
5.933	6.714	7.444	6.854	7.553	9.036	6.987	6.714	9.000	7.499
7.495	7.658	10.097	6.456	7.499	7.499	8.921	8.620	5.731	8.854
7.886	8.000	6.854	7.161	7.208	8.538	8.770	6.854	6.575	7.987
8.495	8.678	6.495	5.933	5.731	5.731	9.036	6.590	7.886	8.824
6.714	7.482	8.000	8.854	7.482	8.432	7.208	8.319	10.143	9.174
8.824	7.499	7.161	7.155	8.678	7.770	6.079	8.000	9.036	8.432
10.602	6.456	6.714	6.893	8.658	5.574	7.921	5.558	7.161	6.762
6.456	8.495	7.553	6.310	7.495	7.482	6.714	7.018	7.499	7.553
6.495	6.818	5.331	9.000	5.574	8.921	7.161	10.143	8.174	7.921
9.000	7.018	8.921	8.319	8.061	7.638	5.331	7.824	8.495	7.161
8.886	7.553	8.237	8.678	8.886	7.155	6.893	6.818	8.886	7.155
9.301	8.174	6.818	6.818	7.131	7.208	8.114	10.602	8.538	7.638
5.176	7.131	8.538	7.921	8.319	5.331	7.495	8.538	7.921	5.886
7.921	5.984	7.161	8.432	6.714	6.575	6.590	8.061	8.432	7.482
9.174	8.237	7.482	5.731	7.658	5.176	8.886	7.161	7.018	5.331
8.854	5.886	7.495	7.499	6.762	5.886	5.558	7.495	6.818	10.143
7.638	5.574	5.558	8.061	8.237	6.854	10.602	8.770	7.208	7.337
8.432	7.770	7.301	8.174	7.886	5.558	7.337	9.301	8.770	7.886
7.987	7.161	7.921	5.886	9.301	5.972	7.770	5.933	5.886	8.770
7.824	6.854	8.658	8.237	8.770	7.553	7.444	9.036	6.987	7.161
6.590	8.854	5.972	5.558	8.824	7.161	7.658	6.854	5.933	7.444
7.770	8.620	8.824	8.538	8.000	5.886	9.301	8.921	7.337	6.590
7.482	8.658	7.337	6.495	5.331	8.174	7.921	8.886	7.770	7.770
